# Comparison of gut viral communities between autism spectrum disorder and healthy children

**DOI:** 10.3389/fcimb.2025.1660970

**Published:** 2025-10-14

**Authors:** Minli Yuan, Qiuxia Wang, Yan Lu, Pan Xu, Chunduo Pan, Wen Zhang, Hongyan Lu

**Affiliations:** ^1^ Department of Pediatrics, The Affiliated Hospital of Jiangsu University, Zhenjiang, China; ^2^ Department of Microbiology, School of Medicine, Jiangsu University, Zhenjiang, China

**Keywords:** autism spectrum disorder, gut viral communities, virome, diversity, viral metagenomics

## Abstract

**Introduction:**

Autism spectrum disorder (ASD) is a complex neurodevelopmental disorder, which brings a great burden to the family and society. Gut microbiota is considered to be an important factor in ASD that easily affects function and development of the immune, metabolic, and nervous systems. However, most available studies have mainly focused on the altered gut bacteria, our knowledge of gut viruses in ASD children remains limited.

**Methods:**

In this study, we collected fecal samples from ASD children and healthy controls, then analyzed and compared the differences of the gut viral communities between the two groups by viral metagenomic techniques.

**Results:**

The alpha diversity of the ASD virome was lower than that of the healthy virome, and the beta diversity had a significant difference between ASD and healthy children. *Podoviridae* accounted for the highest proportion of viruses in ASD patients, while *Alphaflexiviridae* was dominant in healthy controls. There was a statistical difference in the abundance of *Microviridae* between the two groups. Additionally, human astrovirus, picobirnavirus, and norovirus were detected by phylogenetic analysis.

**Discussion:**

This study revealed that alpha diversity was reduced in children with ASD, and different compositions in gut viral communities were observed between ASD patients and healthy controls. Changes in viral diversity and composition deepen our understanding of the differences in the gut viral communities between ASD and healthy children, and also provides a perspective for further exploration of viruses related to ASD children.

## 1 Introduction

Autism spectrum disorder (ASD) is a complex neurodevelopmental disorder, which is characterized by impairments in social communication and interaction, restricted patterns of interest, as well as repetitive and stereotyped behaviours (Lord et al., 2018). The incidence and prevalence of ASD have increased dramatically worldwide over the past decade (Zeidan et al., 2022). Today, ASD is one of the most prevalent neurodevelopmental disorders that affect children (Morris-Rosendahl and Crocq, 2020). It is expected that the number of children with ASD will exceed the number of children with cancer, juvenile diabetes, and pediatric acquired immune deficiency syndrome (AIDS) combined, which can result in serious medical and social problems (Rogge and Janssen, 2019). Frustratingly, little is understood about the etiology of ASD, but it is known that one of the main pathophysiology features of ASD is chronic inflammation in intestinal mucosa (Pape et al., 2019), and increasing evidences indicate that gut microbiota that impacts development and function of the immune, metabolic, and nervous systems may play an important role in ASD (Sharon et al., 2019; Cryan et al., 2020; Zhang et al., 2020; Willyard, 2021).

The gut microbiota includes bacteria, archaea, viruses, fungi, and helminths (Vemuri et al., 2020). Collectively, their respective genomes are called the gut microbiome (Vemuri et al., 2018). Bacteria have gained the most attention in these microorganisms. There is considerable evidence that gut bacteria is closely associated with ASD (Coretti et al., 2018; Luo et al., 2019). Patients with ASD were shown to have decreased abundance of *Bifidobacterium*, *Blautia*, *Dialister*, *Prevotella*, *Veillonella*, and *Turicibacter* (Liu et al., 2019a). Futhermore, decreased *Bacteroidetes*/*Firmicutes* ratio, increased *Lactobacillus* and *Desulfovibrio* species were also present in ASD patients (Tomova et al., 2015). However, merging findings have suggested that as part of the gut microecology, gut virome also appears associated with ASD (Qu et al., 2023; Wan et al., 2024b).

Viruses are abundant in the gut. It is estimated that each gram of human intestinal content includes at least 10^8–^10^10^ virus-like particles(VLPs) (Shkoporov and Hill, 2019). In addition to affecting the digestive system, enteroviruses can also impact the neurological system. At present, several viruses, such as those from the families *Astroviridae*, *Picobirnaviridae*, and *Caliciviridae*, have been reported in some cases of neurological damage (Sato et al., 2016; Gutiérrez-Camus et al., 2022; Deb et al., 2023; Kashnikov et al., 2023). Some enteroviruses can directly infect neurons or glial cells, causing cell damage and death through viral replication and lysis infection, thereby disrupting neural function (Shuid et al., 2021). Furthermore, viral infection can also activate the immune system, leading to an increase in the level of pro-inflammatory cytokines and inducing a systemic inflammatory response, which could affect brain development and thereby cause neurodevelopmental deficits (Meltzer and Van de Water, 2017). A recent report found that there are significant differences in gut-DNA virome abundance and heterogeneity between ASD and typically developing (TD) children (Wan et al., 2024b). In addition, it has been reported that alpha diversity was significantly lower in ASD children especially those who have gastrointestinal symptoms and elevated *Skunavirus* abundance was also observed in children with ASD compared to healthy controls (Qu et al., 2023). However, these studies mainly focus on DNA viruses and do not cover RNA viruses or include analysis of the detected viruses to determine their relationship with previously identified viruses. Overall, in the existing microbiota studies of ASD, the exploration of gut viruses is relatively insufficient.

In recent years, with the advancement of next-generation sequencing technologies, viral metagenomics has been increasingly applied in the study of microbial communities. Different from traditional methods, this technology can rapidly identify all viral sequences in a sample, providing a more comprehensive acquisition of information about the viral community within the sample. It not only expands the information reserve of known viruses but also reveals the characteristics of viruses (Wen et al., 2024).

Therefore, in this study, we further investigated the composition and differences of the gut viral community between ASD patients and healthy controls using viral metagenomic techniques, which provided new insights for us to better understand the impact of gut microecological environment on ASD.

## 2 Materials and methods

### 2.1 Sample collection and preparation

To analyze the gut viral communities between ASD and healthy children, we collected 11 fecal samples from ASD children admitted to the Pediatric Department, Affiliated Hospital of Jiangsu University (Jiangsu Province, China) and 11 fecal samples from healthy children who were in the same hospital for physical examination from September 2023 to March 2024. About 10g of feces were taken from each sample. All ASD patients met the diagnosis standards stipulated in the American Diagnostic and Statistical Manual of Mental Disorders, 5th edition (Posar et al., 2015). Meantime, all participants were required to be no use of antibiotics or antiviral drugs within 3 weeks, no infectious diseases or psychiatric disorders. Subjects with complaints of gastrointestinal symptoms through parent interviews were also excluded. All samples were stored in sterile covered containers and transported to the laboratory via dry ice. In advance of viral metagenomic analysis, each sample was resuspended in 10 volumes of sterile-filtered Dulbecco’s phosphate-buffered saline (DPBS) and vortexed for 5 min vigorously. Then after centrifugation (10 min, 15,000 g, 4°C), the supernatants were collected and stored at -80°C (Wang et al., 2019). Sample collection and all experiments in this study obtained approval from the Ethics Committee of Jiangsu University and the reference number is No.KY2023K0805.

### 2.2 Viral metagenomic analysis

About five hundred microliters of supernatant was drawn from each sample and pooled into the separate sample pool. Sample pools were centrifuged (5 min, 12,000 g, 4°C), and then the supernatant was filtered through a 0.45 μm filter (Millipore) to remove eukaryotic and bacterial cell-sized particles (Conceição-Neto et al., 2015). The filtrate rich in viral particles was treated with DNase and RNase to break down unprotected nucleic acid at 37°C for 60min (Zhang et al., 2014, 2016, 2017). The treatment efficiency of DNase and RNase was validated by quantifying the abundance of the nucleic acid gene via qPCR before and after digestion, which showed a significant reduction. According to the manufacturer’s protocol, total viral nucleic acids were extracted using the QIAamp Viral RNA Mini Kit (Qiagen). Reverse transcription reactions were carried out on these nucleic acid samples composed of DNA and RNA viral sequences with SuperScript III reverse transcriptase and random hexamer primers, followed by a single round of DNA synthesis using Klenow fragment polymerase. All dsDNA products were used for library construction. Then, 22 libraries were constructed using Nextera XT DNA Sample Preparation Kit (Illumina). For bioinformatics analysis, 150-bp paired-end reads generated by NovaSeq were debarcoded using vendor software provided by Illumina (Liu et al., 2016). Clonal reads were removed and low-quality sequencing tails were trimmed using Phred quality score 20 as the threshold. The cleaned reads were then *de novo* assembled by SOAPdenovo2 version r240 using Kmer size 63 with default settings. The assembled contigs and singlets were matched against an in-house viral proteome database using BLASTx with a cut-off E-value of <10^-5^, where the virus BLASTx database was compiled using NCBI virus reference proteome (ftp://ftp.ncbi.nih.gov/refseq/release/viral/) and viral protein sequences from NCBI nr FASTA files (based on annotation taxonomy in Virus Kingdom) (Deng et al., 2015). The selection of the BLASTx E-value cutoff of <10^-5^ could ensured annotation accuracy while effectively controlled the false discovery rate (Skewes-Cox et al., 2014). Candidate viral sequences were further compared to the non-virus non-redundant protein database to eliminate false-positive viral sequences (Kearse et al., 2012). Contigs without significant BLASTx similarity was compared to viral protein families in vFam database using HMMER3 to identify remote viral protein similarities (Johnson et al., 2010; Finn et al., 2011; Skewes-Cox et al., 2014). These BLASTx results generated by DIAMOND (DAA format) were imported into Megan6 software for generation of rma6 format files, which could be further used for subsequent analysis including species accumulation curve, and species rarefaction curve.

### 2.3 Statistical analysis

Utilizing Megan v6.25.7 and R v4.3.2, the statistical analysis related to this study was performed. The composition analysis from 22 libraries was standardized and compared by Megan (Huson et al., 2007). The viral community structure and richness were visualized using the pheatmap and vegan package. Besides, the differences in viral communities were demonstrated using the ggplot2 package and Statistical Analysis of Metagenomic Profiles (STAMP) between the ASD patients group and the healthy controls group. For the multiple-testing involved in the analysis, Benjamini-Hochberg False Discovery Rate (FDR) correction method was adopted. When *P* < 0.05, it was considered to be statistically significant.

### 2.4 Phylogenetic analysis

Phylogenetic analysis was based on predicted nucleotide and protein sequences in this study along with their closest viral relatives that were downloaded from the NCBI GenBank database. Depending on the distance of the evolutionary relationship, for viruses with close kinship, we chose nucleic acid sequences to construct phylogenetic trees, while amino acid sequences were selected for viruses with distant genetic relationship. Related nucleotide and protein sequences were aligned using MUSCLE in MEGA v11.0.13 with default settings (Kumar et al., 2018). Sites which contained more than 50% gaps were temporarily removed from alignments. Bayesian inference trees were then built using MrBayes v.3.2.7 (Ronquist et al., 2012). Moreover, maximum likelihood trees were constructed to confirm all the Bayesian inference trees by MEGA software with 1000 bootstrap replicates (Kumar et al., 2018). Adobe Illustrator and iTOL (https://iTOL.embl.de/) were used to represent the phylogenetic trees.

### 2.5 Quality control

In order to prevent cross-sample contamination and nucleic acid degradation, a robust viral metagenomics pipeline integrated with multiple contamination controls was followed throughout the process. Specifically, sterile ddH_2_O (Sangon Biotech) was prepared and further processed under the same conditions as a blank control to rule out the possibility of nucleic acid contamination. Additionally, all materials that came into direct contact with nucleic acid samples were free of DNase and RNase. DEPC-treated water and RNase inhibitors were used to dissolve nucleic acid samples to preserve nucleic acid integrity.

## 3 Results

### 3.1 Baseline characteristics of ASD and healthy children

The study participants included 11 ASD children and 11 healthy children. The mean age was 5.2 and 4.6 years, respectively. There was no significant statistical difference in age, gender or mode of birth between the two groups (*P* > 0.05) (**Supplementary Table 1**).

### 3.2 Overview of the virome

In the study, after library construction and next generation sequencing on the Illumina NovaSeq platform, the 22 libraries yielded a total of 52,261,771 raw reads with an average percentage of GC content (GC%) of 44.8%. 37,554,145 reads were related to virus in these libraries according to the *de novo* assembled and compared to the GenBank non-redundant protein database using BLASTx (**Supplementary Table 2**). The species richness of all collected samples was revealed by the species rarefaction and accumulation curves (**Supplementary Figure 1**). In majority of 22 libraries, the observed virus species tended to level out at the end, which indicates that the sequencing depth was sufficient to cover all species present in the samples. Even if the sequencing data is increased, the diversity of virus species cannot increase. And the accumulation curves revealed that there may be more than 800 different viruses in 22 libraries.

### 3.3 Diversity and richness of gut virome in ASD and healthy children

**Figure 1:**
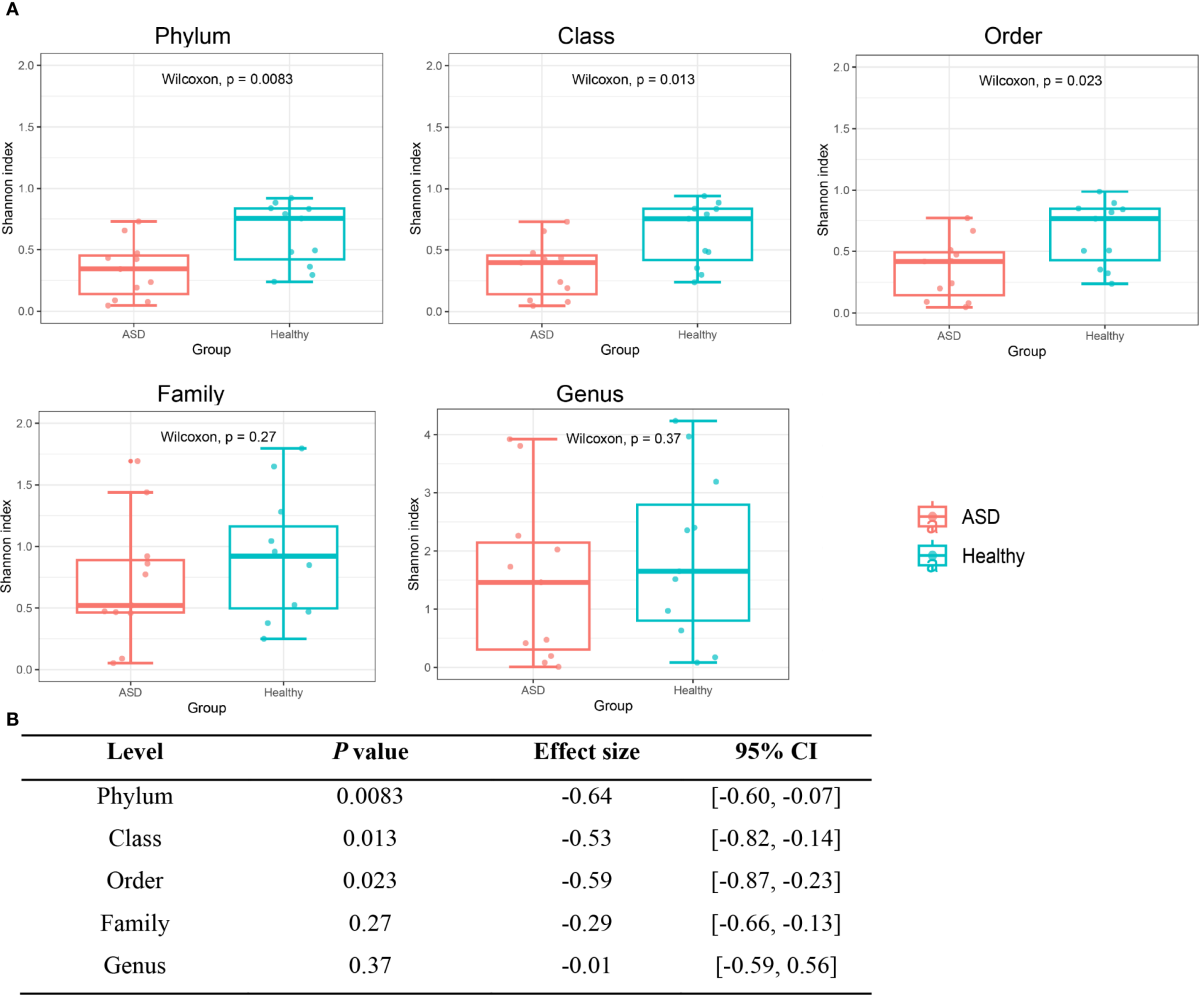
Alpha diversity of the viral communities between ASD patients and healthy controls group at the phylum, class, order, family, and genus levels. **(A)** Comparison of virus alpha diversity with Shannon index between the ASD group and the healthy group at the phylum, class, order, family, and genus levels. The horizontal bars within boxes represent medians. The tops and bottoms of boxes represent the 75th and 25th percentiles, respectively. **(B)** List of *P* values, effect sizes, and confidence intervals at the phylum, class, order, family, and genus levels for the statistical differences.

**Figure 2:**
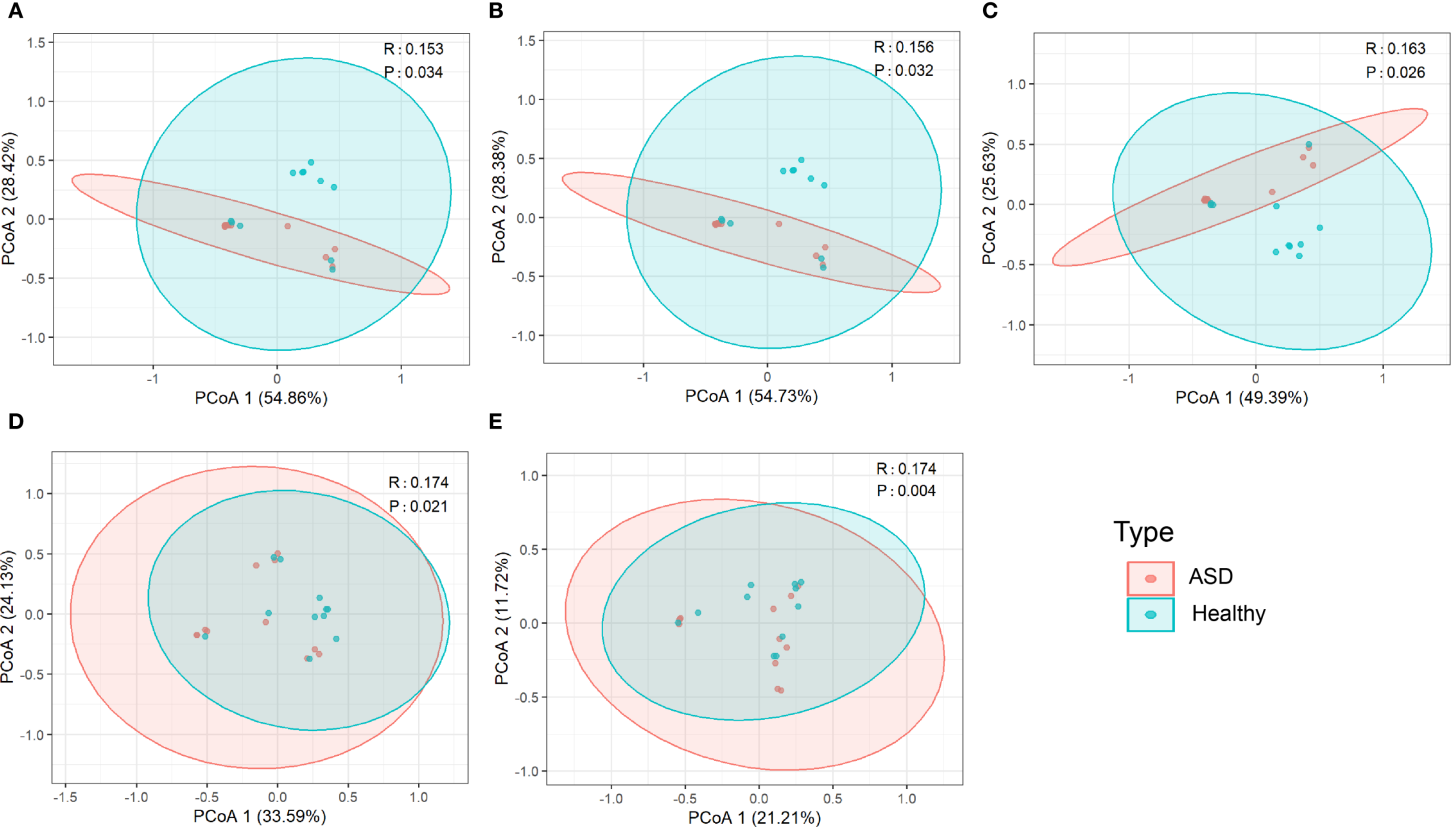
PCoA analysis s of the viral communities between ASD patients and healthy controls group at the **(A)** phylum, **(B)** class, **(C)** order, **(D)** family, and **(E)** genus levels.

To investigate the differences in diversity and richness between the ASD group and the healthy group, alpha diversity analysis and beta diversity analysis were performed on the viral communities from the two groups. In alpha diversity analysis, we could see that α diversity was reduced in children with ASD. It was significantly lower than that in the healthy controls group at the phylum, class, and order levels, but not at the family or genus levels (**Figure 1**). In terms of beta diversity analysis, principal coordinate analysis (PCoA) based on unweighted UniFrac metrics revealed that the composition of the viral communities exhibited statistically significant differences across the two groups at the phylum, class, order, family, and genus levels (**Figure 2**).

### 3.4 Composition and comparison in the viral communities

**Figure 3:**
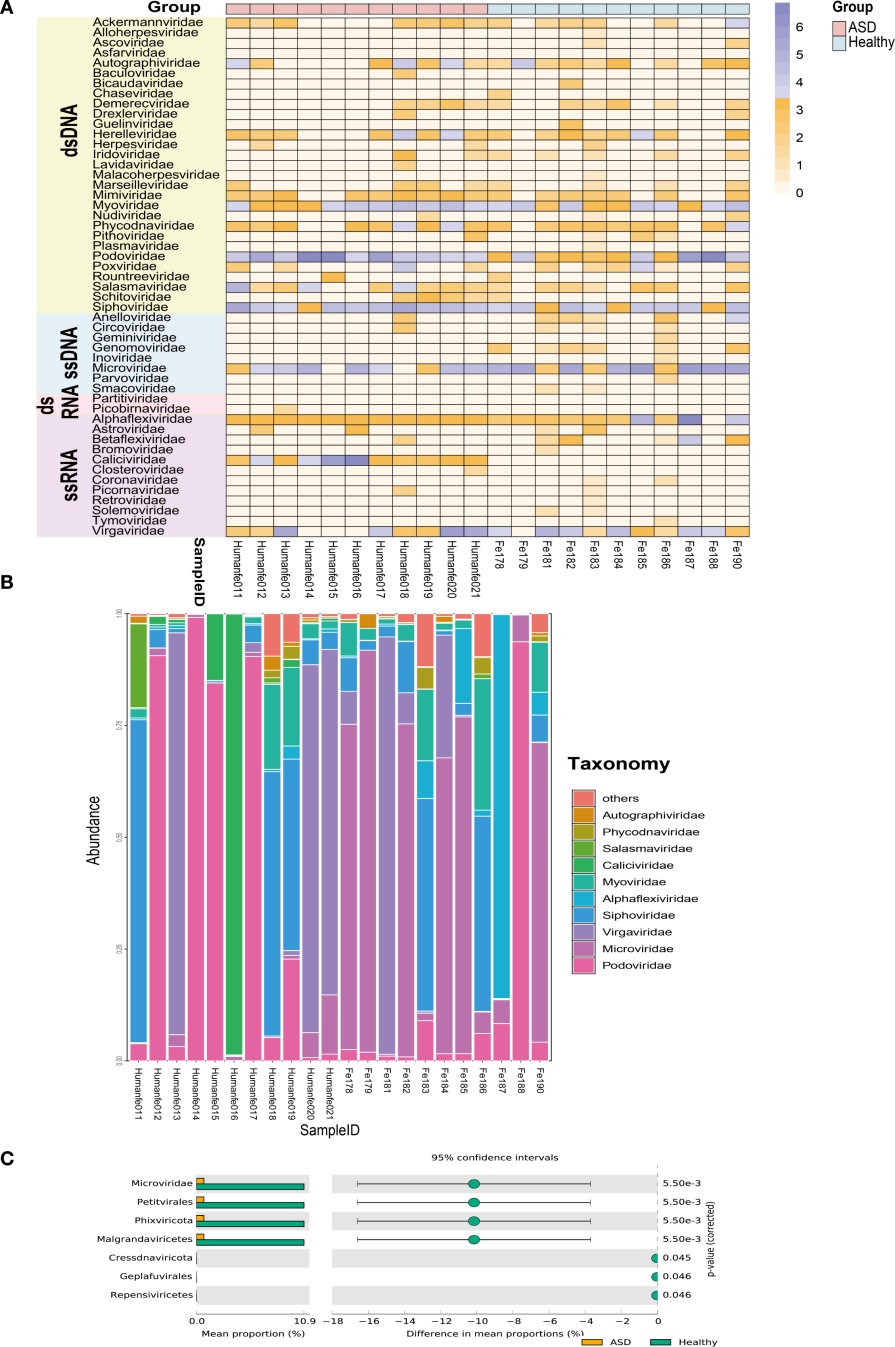
Differentital analysis of the composition of gut viral communities in ASD patients and healthy controls group at the level of family or species. **(A)** Heatmap representing the reads number of each viral family from 22 libraries on a log10 scale. Groups, viral families, and viral types are shown with corresponding colors (see color legend). The read abundance of different viral families in each library are displayed from orange to purple which represent an increasing trend. **(B)** The bar graph showing the relative proportion and taxonomy based on viral families in 22 libraries. **(C)** Analysis of differences between groups using STAMP.

Heatmap at the family level revealed that 51 viral families consisting of 29 double-stranded DNA (dsDNA) viral families, 8 single-stranded DNA (ssDNA) viral families, 2 dsRNA viral families, and 12 ssRNA viral families were present in the 22 libraries (**Figure 3A**). Among them, a total of 18 families showed different compositions (≥2-fold change) between ASD patients and healthy controls (**Supplementary Table 3**). In ASD patients, *Podoviridae* accounted for the highest proportion of viruses, reaching 58.81%, followed by *Caliciviridae* (31.06%), *Virgaviridae* (5.33%), and *Siphoviridae* (2.51%). Each of the family *Microviridae*, *Myoviridae*, and *Salasmaviridae* accounted for more than 1% of viral community compositions. On the other hand, *Alphaflexiviridae* was dominant in the healthy controls group with 50.82%, which was a drastic increase from 0.08% in ASD patients. The next highest proportions of viruses were *Podoviridae* (31.64%) and *Microviridae* (14.46%). For the top ten most abundant viral families between the two groups, we noticed that variations in their proportion in different libraries (**Figure 3B**). To further investigate the differences in the composition of gut viral communities between the ASD group and the healthy group, Statistical Analysis of Metagenomic Profiles analysis was performed on the viral communities from the two groups. According to the results of STAMP analysis, *Phixviricota*, *Malgrandaviricetes*, *Petitvirales*, and *Microviridae* made the greatest contribution to the differences in the healthy control group and the ASD patients group (*P* < 0.01), followed by *Cressdnaviricota*, *Repensiviricetes*, and *Geplafuvirales* (*P* < 0.05) (**Figure 3C**).

### 3.5 Astroviridae

**Figure 4:**
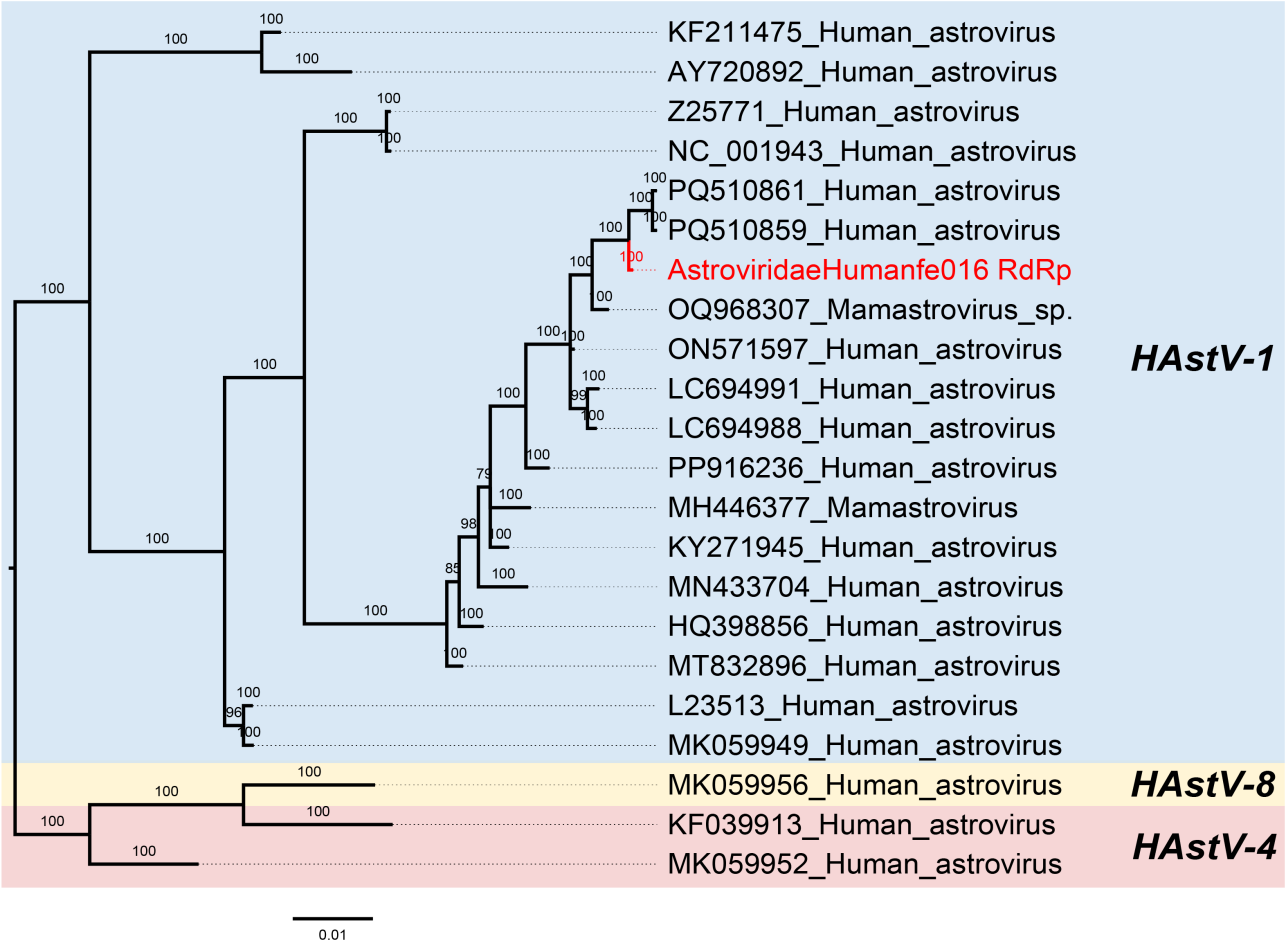
Phylogenetic relationship of *Astroviridae*. The phylogenetic tree is based on nucleic acid sequences of RdRp of viruses belonging to the family *Astroviridae*. Red represents the sequence from this study.

Astroviruses are non-enveloped and single-stranded positive RNA viruses of the *Astroviridae* family with a size of about 6.8-7.9 kb that infect a variety of mammals and avian species (De Benedictis et al., 2011; Marvin et al., 2016). The viral genome contains three overlapping open reading frames (ORFs): ORF1a, ORF1b and ORF2. In astroviruses, ORF1a and ORF1b encode the viral protease and RNA-dependent RNA polymerase (RdRp) respectively, while ORF2 encodes the capsid protein (Bosch et al., 2014). In this study, we obtained a nearly complete astrovirus genome from the library Humanfe016. According to the result of the search in GenBank, it was 99.73% identical to Human astrovirus 1 (GenBank no.PQ510859) collected in Yunnan, China, in 2024. Then, we performed a phylogenetic tree based on the RdRp protein, which indicated AstroviridaeHumanfe016 clustered inside the HAstV-1 genus (**Figure 4**).

### 3.6 Picobirnaviridae

**Figure 5:**
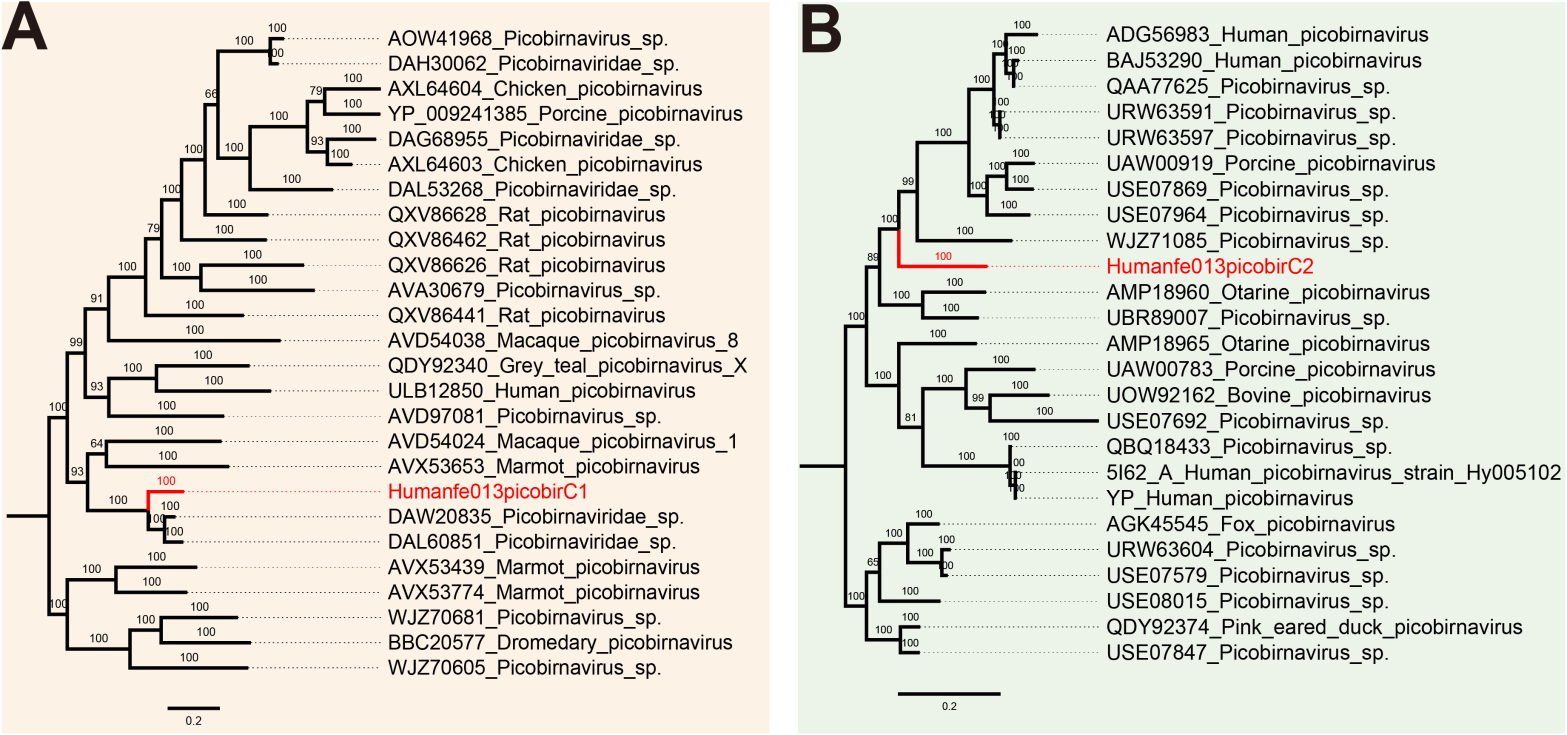
Phylogenetic relationship of *Picobirnaviridae*. Bayesian inference trees were established based on amino acid sequences of capsid protein **(A)** and RdRp **(B)** of *Picobirnaviridae*, within the viruses found in this study are marked with red line.

Picobirnaviruses, the only genus within the family *Picobirnaviridae*, are non-enveloped icosahedral viruses with a diameter of 33–37 nm consisting of two segments (Ganesh et al., 2014). The large segment encodes capsid protein while the small segment encodes RdRp (Xiao et al., 2020). Picobirnaviruses can infect humans and a wide range of animals (Delmas et al., 2019). We identified two strains belonging to *Picobirnaviridae* in this study. In order to analyze the relationship between the newly discovered genome and other known picobirnaviruses, the respective phylogenetic analysis trees were constructed based on the capsid protein (**Figure 5A**) and RdRp (**Figure 5B**), respectively. The results indicated that our sequences clustered with others throughout the phylogenetic trees.

### 3.7 Caliciviridae

Caliciviruses are non-enveloped icosahedral viruses with a single-stranded, positive-sense, polyadenylated RNA genome of 7.4-8.3 kb arranged in two or three major ORFs (Vinjé et al., 2019). Caliciviruses can cause a wide range of diseases in humans and animals, including gastroenteritis, neurological disorders, respiratory infections, and vesicular lesions (Green et al., 2000). The family *Caliciviridae* comprises eleven virus genera, mainly *norovirus*, *lagovirus*, *nebovirus*, *sapovirus*, and *vesivirus* (Lu et al., 2021b). The most important clinical representative is noroviruses.

**Figure 6:**
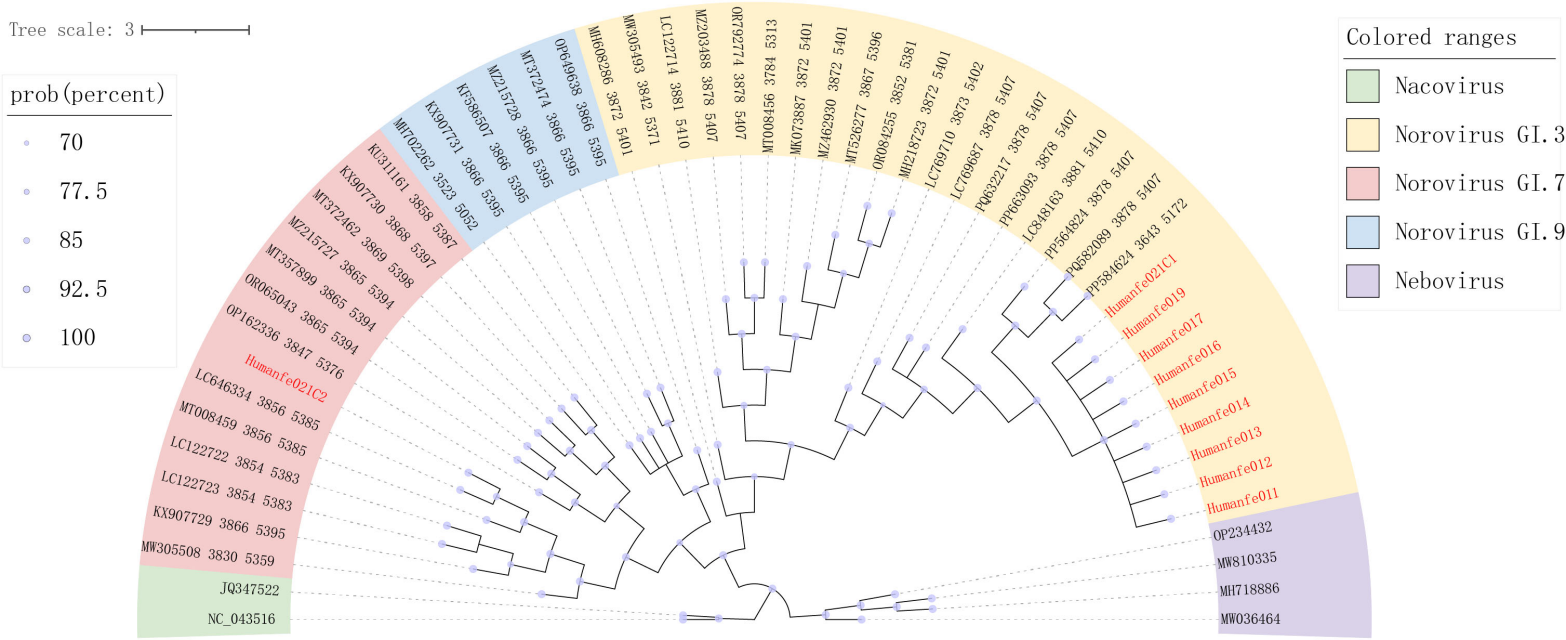
Phylogenetic relationship of *Caliciviridae*. The phylogenetic analysis tree was constructed according to the structure of the RdRp nucleic acid sequences of *Caliciviridae*. Red represents sequences from this study. The scale bar represents the length of the unit representing the value of the difference between organisms or sequences, equivalent to the scale of an evolutionary tree. Each color in the legend represents a genus.

We identified and assembled 6,971,747 reads obtained from sequencing, obtained the genomes of 10 nearly complete caliciviruses through alignment with the NCBI GenBank database using BLASTx data, and then constructed a phylogenetic tree based on the RdRp protein. The phylogenetic tree indicated that caliciviruses in this study had high diversity and were clustered into groups (**Figure 6**). By sequence analysis and phylogeny, nine of these caliciviruses were closely related to genus Norovirus GI.3 which shared more than 99% similarities of their nucleic acid sequences, while Humanfe021C2 and Norovirus GI.7 (Genbank No.LC646334) shared a branch.

## 4 Discussion

In recent years, the worldwide prevalence of ASD has been on the rise (Zeidan et al., 2022), which may be due to the increase in people’s awareness of the disease, the changes in diagnostic criteria, and the increase in the availability of diagnosis (Iglesias-Vázquez et al., 2020). The exact etiopathogenesis of ASD is poorly understood and appear to involve a complex interplay of genetic and environmental factors, of which the gut microbiome is an important environmental factor related to a broad range of neurological and psychiatric disorders and diseases (Kim and Leventhal, 2015; 2019; Cryan et al., 2020; Vemuri et al., 2020). It is reported that the microbiota could shape ASD development and progression by impacting the host defense and inflammatory response (Martin et al., 2018). However, most of the available research literature has focused on describing the altered gut bacteria, there are few studies on the gut virome in the feces of ASD.

In order to compare the gut viral communities between ASD children and healthy controls, we collected fecal samples from children with ASD and healthy controls, then used viral metagenomic analysis to investigate the composition and differences of the gut virome between the two groups in this study. The results showed that there were no differences between ASD and healthy children in terms of age, gender or mode of birth. By depicting the species rarefaction curve and species accumulation curve, the rationality of the sequencing data was illustrated through the species rarefaction curve and species accumulation curve. It should be acknowledged that the inclusion of over 800 different viral species contained in the 22 libraries appears a bit high. This may be because the reads belonging to the same viral genome have different best matches in BLASTx (E-value cutoff of <10^−5^), leading to an overestimation of the species of viruses. To mitigate this, CD-HIT could be used to cluster the assembled contigs and singlets, thereby avoiding redundant annotations from cross-homology.

The human gut virome is characterized by high diversity (Shkoporov et al., 2019). However, compare to healthy children, studies have shown a decrease in the diversity of ASD subjects (Tong et al., 2022; Qu et al., 2023). In our study, alpha diversity was reduced in ASD patients, which was consistent with former studies, and it was significantly lower than that of the healthy controls group in phylum to order level. It is reported that a decrease in microbial diversity is considered to represent in states of disease (Milani et al., 2017). Although the etiology of ASD remains unclear, experimental and clinical studies suggest that ASD is associated with gut microbiota dysbiosis and dysregulation (Zhang et al., 2020; Hughes et al., 2023). Reduced gut viral diversity in children with ASD may create an environment conducive to the overgrowth of pathogenic bacteria and reduced colonization of beneficial bacterial, thereby exacerbating intestinal inflammation (Su et al., 2024; Wan et al., 2024a). Previous studies have linked the loss of gut viral diversity to dysregulation of bacterial hosts and disrupted intestinal barrier function, both of which are pathways implicated in neurodevelopmental alterations associated with ASD via gut-brain axis signaling (Cryan et al., 2020; Rutsch et al., 2020). Thus, our findings, together with existing literature, indicate that reduced gut viral alpha diversity is potentially correlated with ASD susceptibility. Besides, the composition of the viral communities exhibited statistically significant differences in the two groups based on beta diversity.

We also observed that there were differences in the abundance of gut virome between ASD patients and healthy controls, and *Phixviricota*–*Malgrandaviricetes*–*Petitvirale*–*Microviridae* made the greatest contribution to the differences. Recent studies indicated that alterations in the abundance of gut virome may drive gut viral dysbiosis in children with ASD (Wan et al., 2024b; Zeng et al., 2024). *Microviridae* is a small, single-stranded DNA virus that primarily infects bacteria. As one of the common bacteriophages in human intestine, the family *Microviridae* can influence microbial abundance and the composition of microbial communities. By infecting and lysing specific bacterial hosts, *Microviridae* plays a role in regulating the quantity and structure of bacterial populations in the intestinal microecology (Morella et al., 2018). The infecting hosts of this viral family contain *Bacteroides* and *Prevotella* (Reyes et al., 2012). We found a decreased abundance of *Microviridae* in ASD children in our study. Meanwhile, recent research has shown that the amount of *Bacteroides* and *Prevotella* increased in children with ASD compared to the controls (Liu et al., 2019b; Zou et al., 2020). This implies that the weakened lytic regulation effect of such bacteriophages on their host bacteria may reduce the growth inhibition pressure on potential host bacteria, which creates conditions for the increased abundance of the latter. Furthermore, in this study, DNA bacteriophage *Podoviridae* of *Caudovirales* was dominated in the gut virome of ASD children and its abundance is higher compared to healthy children. It is important to note that this observed dominance of *Podoviridae* could be influenced by confounding factors not measured in our study, such as dietary habits or environmental exposures, which may affect the gut virome composition. Therefore, the association between *Podoviridae* and ASD identified here may reflect a broader ecological shift. DNA nature bacteriophages are the most predominant members of the gut virome, which account for 90% of the intestinal viruses (Gregory et al., 2020). Phages have been reported to shape human microbiome diversity and functions through their ability to lyse and kill host bacteria, thereby affecting brain function, mood, and memory (Almand et al., 2017; Fulci et al., 2021; Mayneris-Perxachs et al., 2022; Wu et al., 2022). Wan et al. found an altered gut viral community accompanied by the higher abundance of *Clostridium phage*, *Bacillus phage*,and *Enterobacteria phage* in children with ASD than in healthy individuals (Wan et al., 2024b). Changes in the abundance of these phages are also considered a triggering factor for gut viral ecological system dysbiosis. However, this study used shotgun sequencing, a method that often yields data mixed with a large number of non-viral impurity sequences. This makes it difficult to efficiently and accurately isolate gut viral sequences from the dataset, and the subsequent data analysis work is also extremely cumbersome. The team of Tong reported that *Streptococcal phages* were significantly enriched in the oral phage communities of ASD (Tong et al., 2022). However, the underlying reasons for the enrichment of *Streptococcal phages* in the oral communities of ASD remain unknown. Moreover, many factors, such as narrow food ranges caused by allergies and intolerances, toxic exposures, and poor oral hygiene, may influence the oral microbial environment. Overall, these studies indicate that bacteriophages are potentially associated with the development of ASD.

Moreover, in this study, phylogenetic analysis trees of possible pathogenic viruses were also constructed. Human astroviruses (HAstVs) mainly affect children, elderly people and immunocompromised individuals (Porto et al., 2023). As neurotropic enteroviruses, astroviruses may lead to neuroinflammation and neuronal damage, thereby affecting brain development and function (Arias and DuBois, 2017). Notably, some studies have found that besides enteric diseases, HAstV infections are associated with neurological disorders such as acute encephalopathy (Sato et al., 2016; Lu et al., 2021a; Ghosh et al., 2024). We acquired one strain of astrovirus in the fecal sample of a patient with ASD which shared 99.73% nucleic acid identity to their best match. Based on phylogenetic analysis, the human astrovirus was identified as Human astrovirus 1 (HAstV1). Another virus we found is picobirnavirus which was considered opportunistic enteric pathogens in many species, including humans (Kashnikov et al., 2023). The phylogentic analyses showed that two strains from library Humanfe013 clustered together with other sequences, respectively.

As the major viral enteric pathogen, noroviruses also affect the neurological system in addition to affecting the digestive system (Deb et al., 2023). The presence of norovirus in the central nervous system has been confirmed in the last few years (Kimura et al., 2010; Rocha et al., 2021). Noroviruses can infect brain endothelial cells and increase the expression of matrix metalloproteinases, changing the expression of tight junction proteins and destroying the blood brain barrier (Al-Obaidi et al., 2018). A recent study revealed that they detected human norovirus in stool samples from a patient with seizures and white matter lesions (Gutiérrez-Camus et al., 2022). All calicivirus sequences found in this study were norovirus. Moreover, the noroviruses identified in this study were detected in the ASD group and accounted for a high proportion of the virome, but not in the healthy control group. While this suggests a potential link between norovirus and ASD, this association should be interpreted cautiously. Many confounding factors, including environmental exposure, dietary patterns, genetic background, and immune status, may contribute to norovirus persistence and altered neurological outcomes. Further investigation is needed to determine whether norovirus participates in ASD pathogenesis.

It is worth noting that this study has several limitations. Firstly, the relatively small sample size may not only limit the statistical power and generalizability of the findings but also increase potential false-positive risks. Although sequencing depth was sufficient, the diversity estimates may still be influenced by sample heterogeneity. Secondly, we did not conduct grouping studies on children with ASD in different disease severity and stages, which may also affect gut virome composition. Thirdly, the study only focused on gut viral communities, we did not explore interactions between viruses and other microbiota components such as bacteria, fungi, or archaea. These cross-domain interactions may play a critical role in shaping gut ecology and influencing ASD pathophysiology. The lack of integrated bacteriome data limits the interpretation of virus-bacteria interactions, such as phage-bacterial host dynamics and potential changes in microbial function. In the future, the sample size can be expanded through multi-center research, and group studies can be carried out on children with ASD at different disease degrees and periods to reduce sample deviation and improve the reliability, accuracy and clinical applicability of the research conclusions. Meanwhile, studies on the gut virome and intestinal flora can also be conducted to fill the shortcomings of a single study of viruses or intestinal flora, thereby providing more insights for understanding the mechanisms of ASD and developing new therapies.

In conclusion, this study compared the gut viral communities between ASD patients and healthy controls. Our study revealed a significant difference in the abundance of *Microviridae* between the two groups. The study also confirmed that the alpha diversity of the ASD patients group was lower than that of the healthy controls group, and the beta diversity of the two groups was significantly different. Since there are some limitations, our study deepens the understanding of the differences in the gut viral communities between between ASD and healthy children and contributes to further research on the characteristics and roles of the gut viruses in children with ASD.

## Data Availability

The datasets presented in this study can be found in online repositories. The names of the repository/repositories and accession number(s) can be found in the article/**Supplementary Material**.

## Supplementary material

Supplementary Table 1 Baseline characteristics of ASD and healthy children.

Supplementary Table 2 Library information.

Supplementary Table 3 The summary of the percentage information of viruses at the family level.

Supplementary Figure 1 The diversity of viral species in the 22 libraries. **(A)** Species rarefaction curve drawn in Megan6 software after log-scale transformation. Legends are presented at the bottom of the figure. Each legend represents a library whose color corresponds to the color of the curve. **(B)** Accumulation curve of viral species in viral metagenomes. The X axis represents the number of sampled libraries, and the Y axis represents the cumulative number of species found. The light blue area represents the 95% confidence interval.
