# Supplementary material for: Comparison of gut viral communities between autism spectrum disorder and healthy children
Source: Front Cell Infect Microbiol. 2025 Oct 14;15:1660970. doi: 10.3389/fcimb.2025.1660970 (PMC12558881; doi:10.3389/fcimb.2025.1660970)
Supplement: Supplementary Table 1 — Baseline characteristics of ASD and healthy children. [file Table1.pdf]

**Variable**	**ASD (n=11)**	**Healthy (n=11)**	***P*-value**
Gender			0.658
female	3 (27.3%)	5 (45.5%)	
male	8 (72.7%)	6 (54.5%)	
Delivery mode			1.000
vaginal delivery	5 (45.5%)	6 (54.5%)	
caesarean section	6 (54.5%)	5 (45.5%)	
Age (years)	5.2±1.5	4.6±2.0	0.477
