# Supplementary figures and images for: Comparison of gut viral communities between autism spectrum disorder and healthy children
Source: Front Cell Infect Microbiol. 2025 Oct 14;15:1660970. doi: 10.3389/fcimb.2025.1660970 (PMC12558881; doi:10.3389/fcimb.2025.1660970)

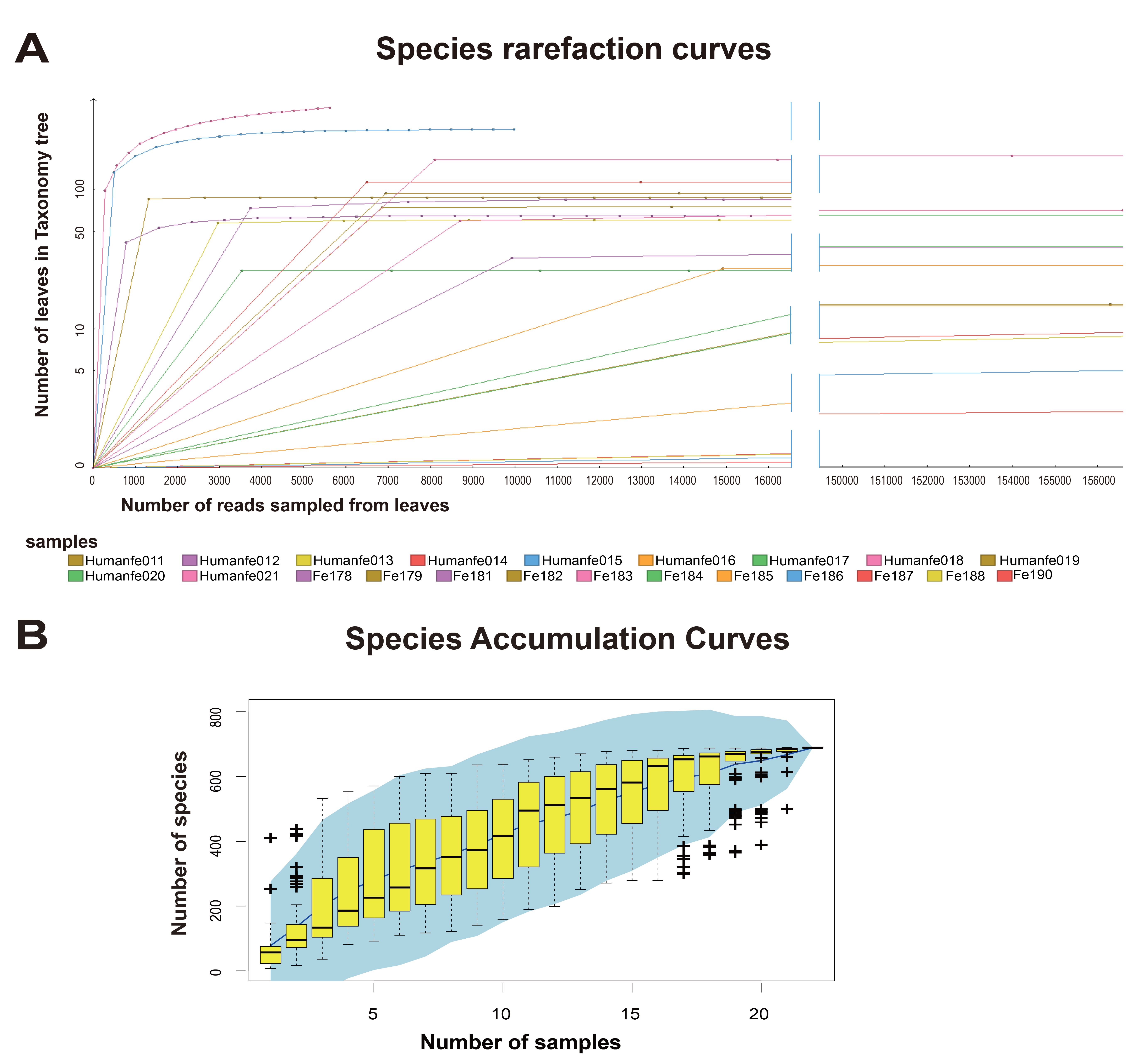

Supplement: Supplementary Figure 1 — The diversity of viral species in the 22 libraries. (A) Species rarefaction curve drawn in Megan6 software after log-scale transformation. Legends are presented at the bottom of the figure. Each legend represents a library whose color corresponds to the color of the curve. (B) Accumulation curve of viral species in viral metagenomes. The X axis represents the number of sampled libraries, and the Y axis represents the cumulative number of species found. The light blue area represents the 95% confidence interval. [file Image1.tif]
